# Supplementary material for: Comparative analysis of infertility healthcare utilization before and after insurance coverage of assisted reproductive technology: A cross-sectional study using National Patient Sample data
Source: PLoS One. 2023 Nov 30;18(11):e0294903. doi: 10.1371/journal.pone.0294903 (PMC10688631; doi:10.1371/journal.pone.0294903)
Supplement: S1 Table — (DOCX) [file pone.0294903.s001.docx]

**S1 Table. Classification of medication.**

| Category | Anatomical Therapeutic Chemical code |
| --- | --- |
| Anesthetics, analgesics, psycholeptics | N01 (N01A, N01B), N02 (N02A, N02B), N05 (N05B, N05C) |
| Antibacterials for systemic use | J01 (J01A, J01D, J01F, J01G, J01M, J01X) |
| Anti-infectives and antiseptics, excl. combinations | G01A |
| Blood substitutes and perfusion solutions | B05 (B05B, B05C, B05X) |
| Drugs for acid-related disorders | A02 (A02A, A02B, A02X) |
| Drugs for functional gastrointestinal disorders | A03 (A03A, A03B, A03F) |
| Drugs used in benign prostatic hypertrophy | G04C |
| Endocrine therapy | L02 (L02A, L02B) |
| Gonadotropins | G03GA |
| Musculo-skeletal system drugs | M (M01A, M03A) |
| Others | A04A, A06A, A07F, A10B, B01A, B02A, B02B, C01C, C07A, M09A, N07A, R03C, R05C, R06A, V03A, V04C, V07A |
| Ovulation stimulants, synthetic | G03GB |
| Sex hormones and modulators of the genital system | G03 (G03B, G03C, G03D) |
| Systemic hormonal preparations, excl. sex hormones and insulins | H (H01C, H02A, H03A) |
| Vitamin B12 and folic acid | B03B |
| X-ray contrast media, iodinated | V08A |
