# Supplementary material for: Comparative analysis of infertility healthcare utilization before and after insurance coverage of assisted reproductive technology: A cross-sectional study using National Patient Sample data
Source: PLoS One. 2023 Nov 30;18(11):e0294903. doi: 10.1371/journal.pone.0294903 (PMC10688631; doi:10.1371/journal.pone.0294903)
Supplement: S2 Table — This information is available on the following website: Korean Statistical Information Service (http://kosis.kr); The price index represents the relative price level of cost adjusted as of 2018. (DOCX) [file pone.0294903.s002.docx]

**S2 Table. Annual average KRW-USD exchange rate and healthcare price index.**

| Year | KRW/USD | Healthcare & medical service price index |
| --- | --- | --- |
| 2016 | 1160.41 | 0.9918 |
| 2018 | 1100.58 | 1.0000 |
| This information is available on the following website: Korean Statistical Information Service (<http://kosis.kr>); The price index represents the relative price level of cost adjusted as of 2018. | | |
