# Supplementary material for: Comparative analysis of infertility healthcare utilization before and after insurance coverage of assisted reproductive technology: A cross-sectional study using National Patient Sample data
Source: PLoS One. 2023 Nov 30;18(11):e0294903. doi: 10.1371/journal.pone.0294903 (PMC10688631; doi:10.1371/journal.pone.0294903)
Supplement: S3 Table — KCD: Korean Standard Classification of Diseases. (DOCX) [file pone.0294903.s003.docx]

**S3 Table. High-frequency comorbidities for female patients.**

| 2016 Female (n = 4,003) | | | | 2018 Female (n = 3,943) | | | |
| --- | --- | --- | --- | --- | --- | --- | --- |
| KCD^a^ code | Disease | No. of patients | Percent | **KCD code** | Disease | No. of patients | Percent |
| K29 | Gastritis and duodenitis | 1,683 | 42.04% | **K29** | Gastritis and duodenitis | 1,752 | 44.43% |
| K05 | Gingivitis and periodontal disease | 1,444 | 36.07% | **K05** | Gingivitis and periodontal disease | 1,600 | 40.58% |
| J20 | Acute bronchitis | 1,440 | 35.97% | **Z31** | Childbirth management | 1,567 | 39.74% |
| J30 | Vasomotor and allergic rhinitis | 1,413 | 35.30% | **J30** | Vasomotor and allergic rhinitis | 1,414 | 35.86% |
| N76 | Other inflammation of the vagina and pudendum | 1,394 | 34.82% | **N76** | Other inflammation of the vagina and pudendum | 1,371 | 34.77% |
| Z34 | Management of normal pregnancy | 1,296 | 32.38% | **Z34** | Management of normal pregnancy | 1,348 | 34.19% |
| N72 | Inflammatory diseases of the cervix | 1,018 | 25.43% | **N72** | Inflammatory diseases of the cervix | 1,071 | 27.16% |
| M54 | Back pain | 840 | 20.98% | **M54** | Back pain | 849 | 21.53% |
| B37 | Candidiasis | 728 | 18.19% | **E28** | Ovary dysfunction | 794 | 20.14% |
| M79 | Other unclassified soft tissue diseases | 682 | 17.04% | **R10** | Abdominal and pelvic pain | 663 | 16.81% |
| J06 | Acute upper respiratory infections of multiple and unspecified sites | 677 | 16.91% | **N91** | Amenorrhea, hypomenorrhea, and oligomenorrhea | 654 | 16.59% |
| J03 | Acute tonsillitis | 669 | 16.71% | **B37** | Candidiasis | 640 | 16.23% |
| N91 | Amenorrhea, hypomenorrhea, and oligomenorrhea | 621 | 15.51% | **J06** | Acute upper respiratory infections of multiple and unspecified sites | 638 | 16.18% |
| R10 | Abdominal and pelvic pain | 620 | 15.49% | **M79** | Other unclassified soft tissue diseases | 633 | 16.05% |
| J04 | Acute laryngitis and tracheitis | 589 | 14.71% | **Z35** | Management of high-risk pregnancy | 616 | 15.62% |
| K21 | Gastroesophageal reflux disease | 550 | 13.74% | **K21** | Gastroesophageal reflux disease | 598 | 15.17% |
| K30 | Functional indigestion | 545 | 13.61% | **Z32** | Pregnancy test | 598 | 15.17% |
| Z35 | Management of high-risk pregnancy | 530 | 13.24% | **J04** | Acute laryngitis and tracheitis | 556 | 14.10% |
| K02 | Dental caries | 513 | 12.82% | **K02** | Dental caries | 547 | 13.87% |
| H10 | Conjunctivitis | 506 | 12.64% | **K30** | Functional indigestion | 533 | 13.52% |
| ^a^KCD: Korean Standard Classification of Diseases | | | | | | | |
