# Supplementary material for: Comparative analysis of infertility healthcare utilization before and after insurance coverage of assisted reproductive technology: A cross-sectional study using National Patient Sample data
Source: PLoS One. 2023 Nov 30;18(11):e0294903. doi: 10.1371/journal.pone.0294903 (PMC10688631; doi:10.1371/journal.pone.0294903)
Supplement: S4 Table — KCD: Korean Standard Classification of Diseases. (DOCX) [file pone.0294903.s004.docx]

**S4 Table. High-frequency comorbidities for male patients.**

| 2016 Male (n = 1,795) | | | | 2018 Male (n = 2,210) | | | |
| --- | --- | --- | --- | --- | --- | --- | --- |
| KCD^a^ code | Disease | No. of patients | Percent | **KCD code** | Disease | No. of patients | Percent |
| K29 | Gastritis and duodenitis | 731 | 40.72% | **K29** | Gastritis and duodenitis | 939 | 42.49% |
| K05 | Gingivitis and periodontal disease | 681 | 37.94% | **K05** | Gingivitis and periodontal disease | 919 | 41.58% |
| J20 | Acute bronchitis | 579 | 32.26% | **Z31** | Childbirth management | 741 | 33.53% |
| J30 | Vasomotor and allergic rhinitis | 555 | 30.92% | **J30** | Vasomotor and allergic rhinitis | 644 | 29.14% |
| M54 | Back pain | 381 | 21.23% | **M54** | Back pain | 483 | 21.86% |
| J06 | Acute upper respiratory infections of multiple and unspecified sites | 296 | 16.49% | **M79** | Other unclassified soft tissue diseases | 368 | 16.65% |
| M79 | Other unclassified soft tissue diseases | 282 | 15.71% | **K21** | Gastroesophageal reflux disease | 324 | 14.66% |
| J03 | Acute tonsillitis | 267 | 14.87% | **J06** | Acute upper respiratory infections of multiple and unspecified sites | 291 | 13.17% |
| K21 | Gastroesophageal reflux disease | 240 | 13.37% | **K02** | Dental caries | 275 | 12.44% |
| K02 | Dental caries | 237 | 13.20% | **S33** | Dislocation, sprain and strain of lumbar spine, pelvis, and ligament | 263 | 11.90% |
| J04 | Acute laryngitis and tracheitis | 219 | 12.20% | **J04** | Acute laryngitis and tracheitis | 243 | 11.00% |
| S33 | Dislocation, sprain and strain of lumbar spine, pelvis, and ligament | 214 | 11.92% | **E78** | Disorders of lipoprotein metabolism and other lipidemia | 233 | 10.54% |
| K30 | Functional indigestion | 210 | 11.70% | **K30** | Functional indigestion | 227 | 10.27% |
| N34 | Urethritis and urethral syndrome | 210 | 11.70% | **A09** | Other gastroenteritis and colitis of infectious and unspecified origin | 222 | 10.05% |
| J02 | Acute pharyngitis | 191 | 10.64% | **L23** | Allergic contact dermatitis | 218 | 9.86% |
| J01 | Acute sinusitis | 174 | 9.69% | **H10** | Conjunctivitis | 194 | 8.78% |
| L23 | Allergic contact dermatitis | 174 | 9.69% | **J01** | Acute sinusitis | 192 | 8.69% |
| H10 | Conjunctivitis | 171 | 9.53% | **K04** | Diseases of pulp and periapical tissues | 185 | 8.37% |
| J00 | Acute rhinopharyngitis [common cold] | 159 | 8.86% | **N41** | Inflammatory diseases of the prostate gland | 180 | 8.14% |
| A09 | Other gastroenteritis and colitis of infectious and unspecified origin | 155 | 8.64% | **J00** | Acute rhinopharyngitis [common cold] | 177 | 8.01% |
| ^a^KCD: Korean Standard Classification of Diseases | | | | | | | |
