# Supplementary material for: Comparative analysis of infertility healthcare utilization before and after insurance coverage of assisted reproductive technology: A cross-sectional study using National Patient Sample data
Source: PLoS One. 2023 Nov 30;18(11):e0294903. doi: 10.1371/journal.pone.0294903 (PMC10688631; doi:10.1371/journal.pone.0294903)
Supplement: S5 Table — (DOCX) [file pone.0294903.s005.docx]

**S5 Table. High-frequency medications for female patients.**

| Category | 2016 | | | | | 2018 | | | | |
| --- | --- | --- | --- | --- | --- | --- | --- | --- | --- | --- |
|  | No. of prescriptions | No. of patients | Total cost | Annual cost per prescription | Annual cost per patient | No. of prescriptions | No. of patients | Total cost | Annual cost per prescription | Annual cost per patient |
| Ovulation stimulants, synthetic | 1,873 | 1,063 | $ 1,895.56 | $ 1.01 | $ 1.78 | 1,754 | 1,063 | $ 1,943.42 | $ 1.11 | $ 1.83 |
| Gonadotropins | 1,868 | 600 | $ 21,371.87 | $ 11.44 | $ 35.62 | 6,835 | 1,534 | $ 400,382.71 | $ 58.58 | $ 261.01 |
| Antibacterials for systemic use | 1,357 | 954 | $ 2,190.24 | $ 1.61 | $ 2.30 | 2,086 | 1,202 | $ 3,391.20 | $ 1.63 | $ 2.82 |
| X-ray contrast media, iodinated | 1,138 | 1,119 | $ 11,997.14 | $ 10.54 | $ 10.72 | 1,134 | 1,118 | $ 13,405.37 | $ 11.82 | $ 11.99 |
| Musculo-skeletal system drugs | 526 | 456 | $ 306.64 | $ 0.58 | $ 0.67 | 767 | 614 | $ 447.50 | $ 0.58 | $ 0.73 |
| Anti-infectives and antiseptics, excl. combinations | 479 | 323 | $ 153.80 | $ 0.32 | $ 0.48 | 446 | 278 | $ 188.79 | $ 0.42 | $ 0.68 |
| Drugs for functional gastrointestinal disorders | 431 | 373 | $ 228.73 | $ 0.53 | $ 0.61 | 484 | 401 | $ 319.98 | $ 0.66 | $ 0.80 |
| Anesthetics, analgesics, psycholeptics | 290 | 182 | $ 317.21 | $ 1.09 | $ 1.74 | 1,688 | 692 | $ 2,206.47 | $ 1.31 | $ 3.19 |
| Sex hormones and modulators of the genital system | 273 | 147 | $ 1,093.90 | $ 4.01 | $ 7.44 | 891 | 445 | $ 5,459.59 | $ 6.13 | $ 12.27 |
| Others | 231 | 173 | $ 138.39 | $ 0.60 | $ 0.80 | 813 | 485 | $ 280.80 | $ 0.35 | $ 0.58 |
| Blood substitutes and perfusion solutions | 227 | 149 | $ 323.07 | $ 1.42 | $ 2.17 | 1,423 | 669 | $ 1,817.77 | $ 1.28 | $ 2.72 |
| Systemic hormonal preparations, excl. sex hormones and insulins | 178 | 120 | $ 67.23 | $ 0.38 | $ 0.56 | 1,695 | 641 | $ 66,020.82 | $ 38.95 | $ 103.00 |
| Vitamin B12 and folic acid | 90 | 75 | $ 193.55 | $ 2.15 | $ 2.58 | 118 | 98 | $ 272.16 | $ 2.31 | $ 2.78 |
| Drugs for acid related disorders | 53 | 50 | $ 17.22 | $ 0.32 | $ 0.34 | 99 | 74 | $ 25.68 | $ 0.26 | $ 0.35 |
| Endocrine therapy | 18 | 12 | $ 298.54 | $ 16.59 | $ 24.88 | 710 | 428 | $ 17,114.61 | $ 24.11 | $ 39.99 |
