# Supplementary material for: Comparative analysis of infertility healthcare utilization before and after insurance coverage of assisted reproductive technology: A cross-sectional study using National Patient Sample data
Source: PLoS One. 2023 Nov 30;18(11):e0294903. doi: 10.1371/journal.pone.0294903 (PMC10688631; doi:10.1371/journal.pone.0294903)
Supplement: S6 Table — (DOCX) [file pone.0294903.s006.docx]

**S6 Table. High-frequency medications for male patients.**

| Category | 2016 | | | | | 2018 | | | | |
| --- | --- | --- | --- | --- | --- | --- | --- | --- | --- | --- |
|  | No. of prescriptions | No. of patients | Total cost | Annual cost per prescription | Annual cost per patient | No. of prescriptions | No. of patients | Total cost | Annual cost per prescription | Annual cost per patient |
| Antibacterials for systemic use | 165 | 95 | $ 1,502.81 | $ 9.11 | $ 15.82 | 226 | 146 | $ 1,614.62 | $ 7.14 | $ 11.06 |
| Others | 92 | 24 | $ 192.85 | $ 2.10 | $ 8.04 | 66 | 25 | $ 69.88 | $ 1.06 | $ 2.80 |
| Anesthetics, analgesics, psycholeptics | 84 | 25 | $ 338.76 | $ 4.03 | $ 13.55 | 92 | 27 | $ 222.33 | $ 2.42 | $ 8.23 |
| Blood substitutes and perfusion solutions | 65 | 22 | $ 121.57 | $ 1.87 | $ 5.53 | 62 | 24 | $ 129.55 | $ 2.09 | $ 5.40 |
| Musculo-skeletal system drugs | 57 | 39 | $ 181.23 | $ 3.18 | $ 4.65 | 43 | 33 | $ 81.29 | $ 1.89 | $ 2.46 |
| Drugs for acid related disorders | 50 | 35 | $ 174.62 | $ 3.49 | $ 4.99 | 36 | 30 | $ 90.94 | $ 2.53 | $ 3.03 |
| Drugs for functional gastrointestinal disorders | 28 | 22 | $ 30.99 | $ 1.11 | $ 1.41 | 38 | 26 | $ 45.34 | $ 1.19 | $ 1.74 |
| Gonadotropins | 7 | 3 | $ 116.88 | $ 16.70 | $ 38.96 | 46 | 4 | $ 1,281.79 | $ 27.87 | $ 320.45 |
| Drugs used in benign prostatic hypertrophy | 6 | 3 | $ 173.04 | $ 28.84 | $ 57.68 | 4 | 4 | $ 65.73 | $ 16.43 | $ 16.43 |
| Sex hormones and modulators of the genital system | 5 | 2 | $ 290.82 | $ 58.16 | $ 145.41 | - | - | - | - | - |
| Systemic hormonal preparations, excl. sex hormones and insulins | 4 | 3 | $ 2.85 | $ 0.71 | $ 0.95 | 2 | 2 | $ 2.32 | $ 1.16 | $ 1.16 |
